# A mouse model for X-linked Alport syndrome induced by Del-ATGG in the *Col4a5* gene

**DOI:** 10.3389/fmed.2023.1086756

**Published:** 2023-03-08

**Authors:** Wei-qing Wu, Jia-xun Zhang, Ying-xia Cui, Ming-chao Zhang, Xiao-hang Chen, Shan Duan, Cai-hong Zeng, Pei-ning Li, Xiao-jun Li

**Affiliations:** ^1^Institute of Clinical Laboratory Science, Jinling Hospital, The First School of Clinical Medicine, Southern Medical University, Nanjing, China; ^2^Center of Medical Genetics, Shenzhen Maternity and Child Healthcare Hospital, The First School of Clinical Medicine, Southern Medical University, Shenzhen, China; ^3^National Clinical Research Center of Kidney Diseases, Jinling Hospital, The First School of Clinical Medicine, Southern Medical University, Nanjing, China; ^4^The Genetics Laboratory, Longgang District Maternity and Child Healthcare Hospital of Shenzhen City, Shenzhen, Guangdong, China; ^5^Laboratory of Molecular Medicine, Institute of Maternal and Child Medicine, Shenzhen Maternity and Child Healthcare Hospital, Southern Medical University, Shenzhen, China; ^6^Department of Genetics, Yale School of Medicine, New Haven, CT, United States

**Keywords:** X-linked Alport syndrome, mouse model, *Col4a5 gene*, frameshift variant, RNA-sequencing

## Abstract

Alport syndrome (AS) is an inherited glomerular basement membrane (GBM) disease leading to end-stage renal disease (ESRD). X-linked AS (XLAS) is caused by pathogenic variants in the *COL4A5* gene. Many pathogenic variants causing AS have been detected, but the genetic modifications and pathological alterations leading to ESRD have not been fully characterized. In this study, a novel frameshift variant c.980_983del ATGG in the exon 17 of the *COL4A5* gene detected in a patient with XLAS was introduced into a mouse model in by CRISPR/Cas9 system. Through biochemical urinalysis, histopathology, immunofluorescence, and transmission electron microscopy (TEM) detection, the clinical manifestations and pathological alterations of Del-ATGG mice were characterized. From 16 weeks of age, obvious proteinuria was observed and TEM showed typical alterations of XLAS. The pathological changes included glomerular atrophy, increased monocytes in renal interstitial, and the absence of type IV collagen α5. The expression of *Col4a5* was significantly decreased in Del-ATGG mouse model. Transcriptomic analysis showed that differentially expressed genes (DEGs) accounted for 17.45% (4,188/24003) of all genes. GO terms indicated that the functions of identified DEGs were associated with cell adhesion, migration, and proliferation, while KEGG terms found enhanced the degradation of ECM, amino acid metabolism, helper T-cell differentiation, various receptor interactions, and several important pathways such as chemokine signaling pathway, NF-kappa B signaling pathway, JAK–STAT signaling pathway. In conclusion, a mouse model with a frameshift variant in the *Col4a5 gene* has been generated to demonstrate the biochemical, histological, and pathogenic alterations related to AS. Further gene expression profiling and transcriptomic analysis revealed DEGs and enriched pathways potentially related to the disease progression of AS. This Del-ATGG mouse model could be used to further define the genetic modifiers and potential therapeutic targets for XLAS treatment.

## 1. Introduction

Alport syndrome (AS) is the most common inherited glomerular disease caused by pathogenic variants of the *COL4A3*, *COL4A4*, or *COL4A5* genes that encode type IV collagens (1, 2). X-linked Alport syndrome (XLAS) is more severe than autosomal dominant form, which caused by pathogenic variants in the *COL4A5* gene (3). Most men and 15–30% of women with XLAS develop kidney failure, the age of them progress to end-stage renal disease (ESRD) generally were 20 years and 60 years, respectively, (4, 5). The pathogenic variants were detected in many AS patients, but the underlying mechanisms of progression to ESRD has not been fully elucidated.

Animal models of AS have been used to explore the disease mechanisms and to experiment effective therapeutic strategies (6). Several AS animal models including mouse, rat and canine have been developed, while there are only two XLAS mouse models were reported, one with a G5X variant in exon 1 and another with a R471X variant in exon 21 of the *Col4a5* gene (7–9). Given the severity of clinical manifestations varies according to the types of pathogenic variants in the *Col4a5* gene, mouse models of other variant types need to be further explored (10).

High-throughput transcriptome sequencing has unique advantages in understanding the molecular mechanisms of disease through the identification of differentially expressed genes (DEGs) and potential biomarkers that affect the disease progression. There were two previous studies on transcriptome alterations in XLAS, one used RNA sequencing to identify DEGs in the renal tissues of XLAS dogs and another used microarray technology to detect DEGs in the renal cortex of XLAS dogs (11, 12). The gene expression profiles of the XLAS mouse have not been analyzed previously.

The clustered regularly interspaced short palindromic repeats/CRISPR-associated (CRISP/Cas) system has been effective in genome editing to generate mouse models carrying pathogenic variants (13). In our previous study, a novel frameshifting deletion c.980_983del ATGG (p.D327Vfs*18) in exon 17 of the *COL4A5* gene was identified in an XLAS patient. We used the CRISPR/Cas9 system to generate a mouse model with this frameshift deletion in the *Col4a5* gene. We performed biochemical, histologic, gene expression, and transcriptomic analyses to understand the molecular mechanisms from this XLAS mouse model.

## 2. Materials and methods

### 2.1. Clinical findings of the patient

**Figure 1:**
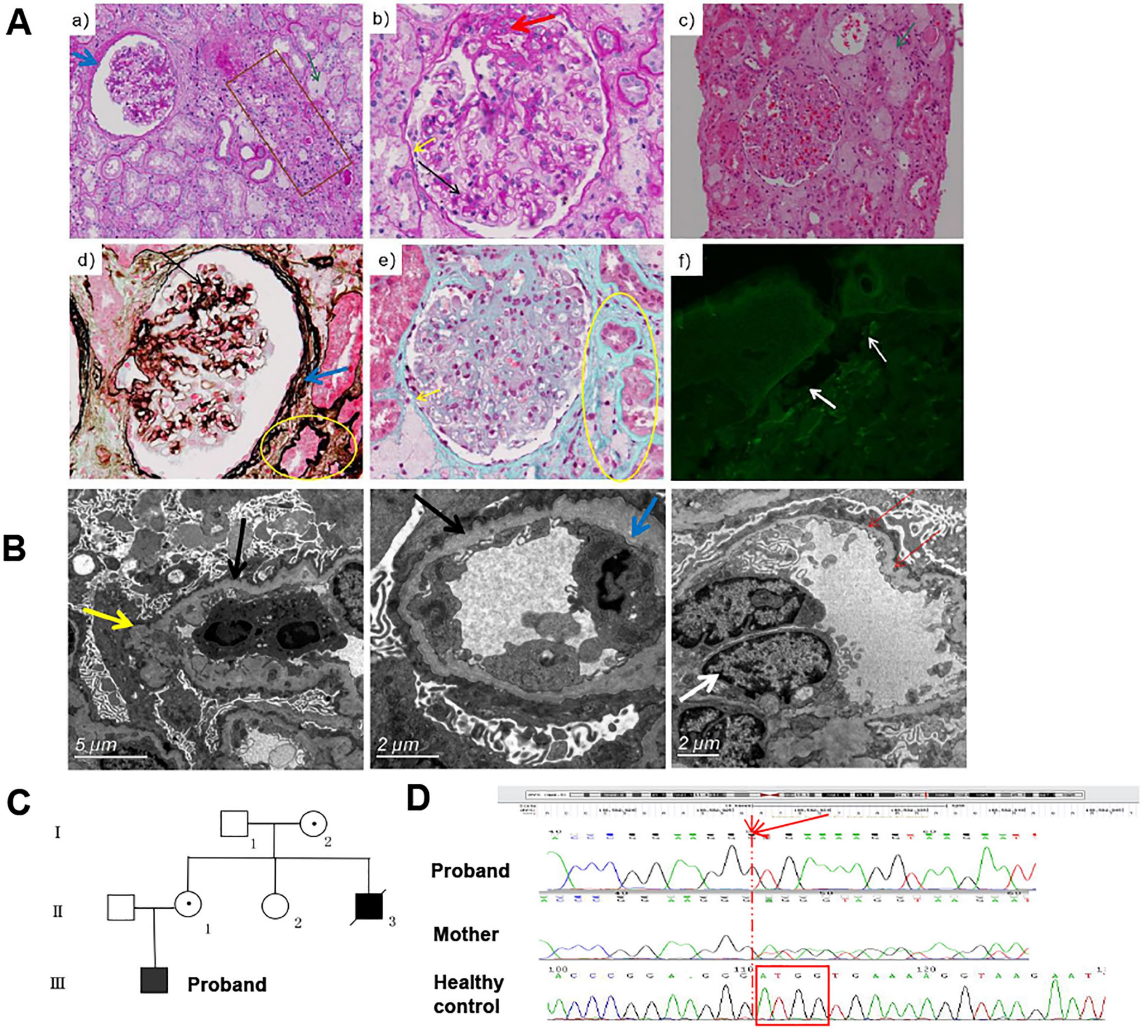
The clinical information and pathological manifestations of a XLAS patient with c.980_983delATGG variant in *COL4A5* gene. **(A)** Histological alterations in kidney tissues of the patient. **a,b,** PAS staining (×200, ×400); **c,** H&E staining (×200); **d,** PASM staining (×400); **e,** Masson staining (×400);**f,** immunofluorescence of type IV collagen α5 chain. The black arrow indicated the mildly to moderately widened mesangial area and mononuclear cells infiltration. The yellow arrow indicated the adhered loops. The blue arrow indicated the increased and stratified parietal layer. The green arrow indicated the clustered foam cells. The brown box indicated the atrophy and thickening renal tubule. The yellow circle indicated the focal fibrosis. The white arrow indicated the position of BM. **(B)** TEM results of the patient. Black arrows indicate GMB, with diffuse and irregular thinning and thickening; inflammatory cells infiltrate in the loops, occasionally endothelial cells are paired (white arrows); the inner and outer loose layers are irregular, and local swelling (yellow arrow). The blue arrow indicated the fusion (30–40%) of the mesangial region and podocyte foot process effacement. **(C)** The pedigree diagram of the patient. **(D)** The Sanger sequencing results of the proband and his mother.

The male patient was the only child from a non-consanguineous couple. At the age of three years, he was noted with an abnormal urine test. He was hospitalized at the age of 15 years due to renal disease. The blood examination revealed normal results except for low value of total plasma protein 50.4 g/L [normal range 64-83 g/L] and Globulion 13.4 [normal range 20–30 g/L]. Urinalysis showed 6.88 g [normal range ≤ 0.4 g] of 24 h urine protein and microscopic hematuria. Light microscopy examination of kidney biopsy showed five globally sclerosed glomerulus among 11 glomeruli, mild to moderate expansion of mesangial with increased mesangial matrix, swelling and twisted glomerular capillary loops with some adhered to the thickened and layered parietal layer of glomerular capsule, interstitial inflammation, and significant tubular and interstitial fibrosis (Figure 1A). Immunofluorescence test showed that α5 chain of collagen IV was absent in skin tissue of the patient (Figure 1A). The α3 and the α5 chain of collagen IV were absent in renal tissue of the patient (picture not provided). Electron microscopy examination of kidney biopsy revealed diffuse thicked, thinned, and lamellated glomerular basement membrane (GBM) with a basket-weave appearance. Foot process effacement was prominent (Figure 1B). The patient was diagnosed with AS. As the renal function deteriorated, the patient was recommend to undergo renal transplantation at the age of 23 years. His mother (II2) and uncle (II4) suffered from chronic nephritis, and the uncle died of renal failure at the age of 18 years (Figure 1C).

Genetic testing was performed on DNA extracted from peripheral blood of this patient. A novel frameshifting deletion c.980_983del ATGG (p.D327Vfs*18) in exon 17 of the *COL4A5* gene was identified by next-generation sequencing and verified by Sanger sequencing (Figure 1D). This variant was classified as pathogenic according to the ACMG guidelines with evidence supporting that the deletion may cause nonsense-mediated mRNA decay (NMD) as a frameshift changes in the open reading frame (14, 15).

### 2.2. Animals

The C57BL/6J mice were purchased from GemPharmatech Co. Ltd. (Nanjing, China). C57BL/6J mice were provided with a standard chow diet and water, housed in a temperature- and humidity-controlled room. All animal experiments were approved by the Ethics Committee of Institutional Animal Care and Use Committee of Nanjing University School of Medicine.

### 2.3. Generation of *Col4a5* Del-ATGG knock-in mouse model

**Figure 2:**
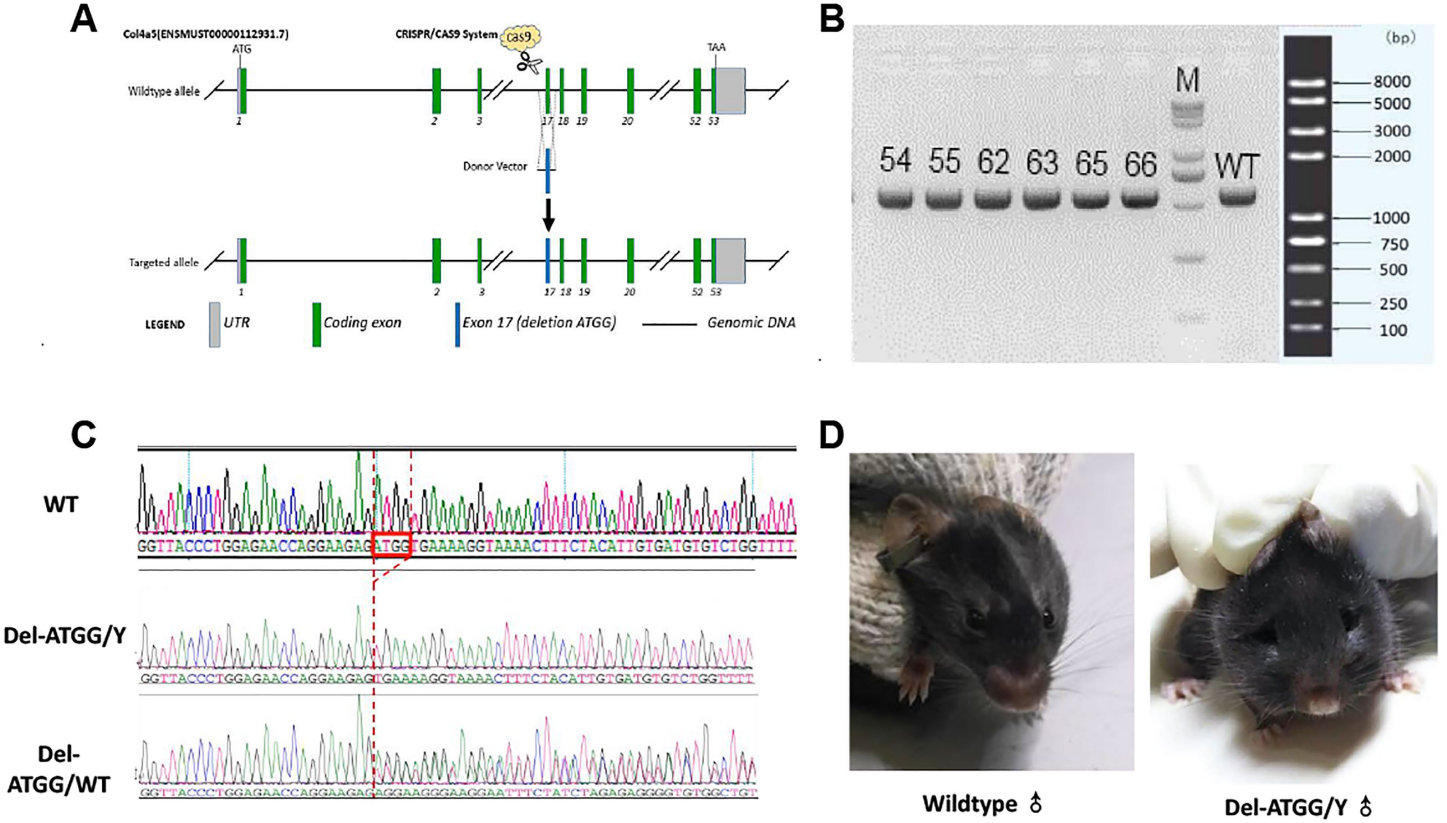
Targeting strategy, genotyping and clinical appearance of Del-ATGG mice. **(A)** The target site was genetically modified through homologous recombination in embryonic stem cells using CRISPR/Cas9 system. **(B)** The electrophoresis results of PCR products in the target region of F1 mice. **(C)** Sequencing results of the target region of the F1 generation mouse. **(D)** The representative images of appearance of wild-type and Del-ATGG mice at 28 weeks of age. 54–66, number of F1 generation mice; WT, wild-type control; N, blank control; M, DNA molecular marker; Del-ATGG/Y, hemizygous male mice (ATGG base deletion); Del-ATGG/wt, heterozygous female mice. The red box is the position of ATGG deletion, the red dotted line indicates the position of deletion of different samples.

Transcript mouse *Col4a5–*202 (ENSMUST00000112931.7) was selected for presentation of our strategy. The *Col4a5*-del ATGG knock-in mice were constructed *via* CRISPR/Cas9 system (Figure 2A). Firstly, a sgRNA harboring the pathogenic sites in exon 17 of the *Col4a5* (sgRNA sequence: ACCTTTTCACCATCTCTTCC) and the donor vector with the deletion variant was designed and constructed *in vitro*. Then, Cas9 mRNA, sgRNA, and donor vector were co-injected into zygotes of C57BL/6J background. Thereafter, the zygotes were transferred into the oviduct of pseudopregnant ICR females. F0 mice were birthed after 19 ~ 21 days of transplantation, all the offspring of *Col4a5*-del ATGG females (F0 mice) were identified by PCR and Sanger sequencing.

The heterozygous female mice (including F0 generation) were bred with C57BL/6J wild-type male mice, all offspring were genotyped to select the heterozygous female mice and hemizygous male mice. The health status was regularly observed, focusing on hair change and appearance of edema.

### 2.4. Genotyping

According to the manufacturer’s protocol, the genomic DNA was extracted from the peripheral blood cells in the ear vein of mice using the Genomic DNA Kit (TIANGEN, China). The primers used in this assay were as follows: *Col4a5*-wt-tF1, 5’-CCTTCTTCTGAATGTTCATATCCAGG-3′ and *Col4a5*-wt-tR1, 5’-CACTGGAATAGAGATCAAACCTCCTC-3′ (product size: 584 bp); PCR was performed using 2 × Taq Master Mix, Dye Plus (Vazyme, China). The cycle conditions were as follows: 98°C for 30 s, 60°C(−0.5°C/cycle) for 30 s, and 72°C for 45 s for 20 cycles, and 98°C for 30 s, 55°C for 30 s, and 72°C for 45 s for 20 cycles. PCR products were separated by electrophoresis on 1.5% agarose gels, the range of DNA ladder was 100–8000 bp (GenStar, China). Direct Sanger sequencing of the PCR products was performed on an ABI 3730 DNA analyzer.

### 2.5. Biochemical analysis of blood and urine

The 24-h urine of mice was collected every 4 weeks from the age of 16 weeks. Urine biochemical parameters including albumin (ALB), creatinine (CRE), and albumin-to-creatinine ratio (ACR) were analyzed by the automatic biochemical analyzer (Hitachi7600, Japan) after centrifuged at 3000 rpm for 20 min. Serum ALB was also analyzed by automatic biochemical analyzer. The quantitative assay of urine ALB was immunoturbidimetric method. The urine CRE assay was based on L-Type Creatinine M principle (HMMPS).

### 2.6. Detection of histological changes of kidney

To detect the histological changes of the Del-ATGG mouse model, the renal tissues of 30 weeks of Del-ATGG and wild-type male mice were dissected for histology, transmission electron microscopy (TEM), and immunofluorescence assay.

#### 2.6.1. Histology

Renal tissues were fixed in 10% neutral formaldehyde for making paraffin-embedded sections. Hematoxylin and eosin (H&E), periodic acid-Schiff (PAS), and Masson staining were performed as previously described (14). The staining results were assessed in a series of randomly selected ten high-power fields. The stained slides were reviewed independently by two observers blinded to the genotype of mice.

#### 2.6.2. Tem

Renal tissues were cut into 0.5 ~ 1 mm^3^ pieces and pre-fixed with 4% glutaraldehyde at 4°C temperature. Then, the tissues were fixed in 1% osmium tetroxide for 2 h. After gradient dehydration of acetone, the tissues were embedded in Epon 812 and prepared for ultrathin sections. The sections were washed three times with CO_2_-free hydrogen peroxide, stained with uranyl acetate for 30 min, then washed by deionized water.

#### 2.6.3. Immunofluorescence assay

Frozen renal tissue sections were fixed in 10% neutral formaldehyde for 15 min, permeabilized with 0.5% Triton X-100 and blocked for 30 min with 10% BSA. The sections were incubated with anti-collagen IV α5 antibody (Sigma) for 1 h. The stained sections were assessed under a fluorescence microscope.

### 2.7. mRNA expression analysis of collagen genes

The mRNA expression analysis was performed to assess the severity of the disease with progressive manifestation. Since the renal manifestation of 28-week-age mice was end-stage renal disease, the comprehensive manifestation of renal tissues could be observed. Three Del-ATGG and three wild-type male mice with the age of 28-weeks were selected. Total RNA was extracted from the renal tissues using RNA easy fast kit (TIANGEN, China). Reverse transcription reaction and real-time quantitative PCR (qPCR) was performed with One-Step RT-qPCR Kit (Accurate biology, China). Then, the expression of the type IV collagen α1-6 (*Col4a1–6*) genes was detected. The primer sequences are listed in Supplementary Table S1. The qPCR result was compared with the expression data of sequencing.

### 2.8. RNA-sequencing and differentially expressed genes analyses

In order to explore differential expression of genes (DEGs) in renal tissue under severe condition of Alport syndrome, 28-week-age mice were selected for RNA-seq, as the mice in this period were at the end stage of renal disease.

#### 2.8.1. RNA-seq

Total RNA was extracted from renal tissues of four Del-ATGG and four wild-type male mice with 28-week-age using RNA easy fast kit. The transcriptomic sequencing was performed by Novogene Co., Ltd. (China). The data that support the findings of this study have been deposited into CNGB Sequence Archive (CNSA) of China National GenBank DataBase (CNGBdb) with accession number CNP0002438. The R program (version 3.5.1) was used to process raw data, and the R package DESeq2 was used to identify DEGs between Del-ATGG and wild-type mice. DESeq2 returned a *p-*value determined by Wald statistics, the Benjamini-Hochberg method was used to determine the false discovery rate (FDR) for multiple comparison testing. DEGs were defined as gene expression significantly different (log_2_ |fold change| > 1) with a FDR less than 5% (Q < 0.05) between different cohorts. The determining and plotting in the heatmap were calculated by the log2 (absolute_expression/median expression of wild-type mice) per gene. The normalized data were log2 transformed and median centered, and the expression value “zero” was set to the overall minimum value.

The expression of several DEGs was detected by qPCR to assess the reliability of the RNA-seq results. The primer sequences for validation are also listed in Supplementary Table S1. Additionally, the read counts of sequences in the upstream and downstream of the *Col4a5* gene ATGG-del site were screened carefully to explore the alteration in the full length of transcripts and to evaluate whether NMD mechanism act on the expression alteration.

#### 2.8.2. Gene ontology and Kyoto encyclopedia of genes and genomes pathway analyses

To evaluate the molecular mechanism of identified DEGs, gene set enrichment analysis was performed to define gene function. DEGs that were significantly over-represented (log_2_ |foldchange| > 1) were identified with the functional annotation tool DAVID. Annotated genes were then clustered into biological processes and KEGG pathways using Gene ontology and KEGG pathway analyses.

#### 2.8.3. Statistical analyses

Statistical analyses were performed using SPSS (SPSS Inc., IL) and GraphPad Prism (GraphPad Software Inc., CA) software. Comparing results from two groups were carried out using the Student’s *t*-test. All statistical tests were two-sided. Differences were considered statistically significant at *p* values <0.05. Experiments were performed at least three times.

## 3. Results

### 3.1. Generation of Del-ATGG mice

All F0 generations mice were generated using the CRISPR/Cas9 system as described above. Five Del-ATGG heterogeneous female mice confirmed by Sanger sequencing were obtained (Figures 2B,C). By mating the heterozygous female with wild-type male mice, sufficient hemizygous and heterozygous mice were generated from subsequent passages.

Daily observation of hemizygous mice showed abnormal performances such as weight loss, rough hair, edema, and foamy urine appeared by 20 weeks. These abnormal performances were gradually progressed, and obvious edema usually appeared by 28 weeks (Figure 2D). The average survival time of Del-ATGG hemizygous mice was 32 weeks, and almost all of them died before 40 weeks (data not showed).

### 3.2. Biochemical analysis of blood and urine of Del-ATGG mice

**Figure 3:**
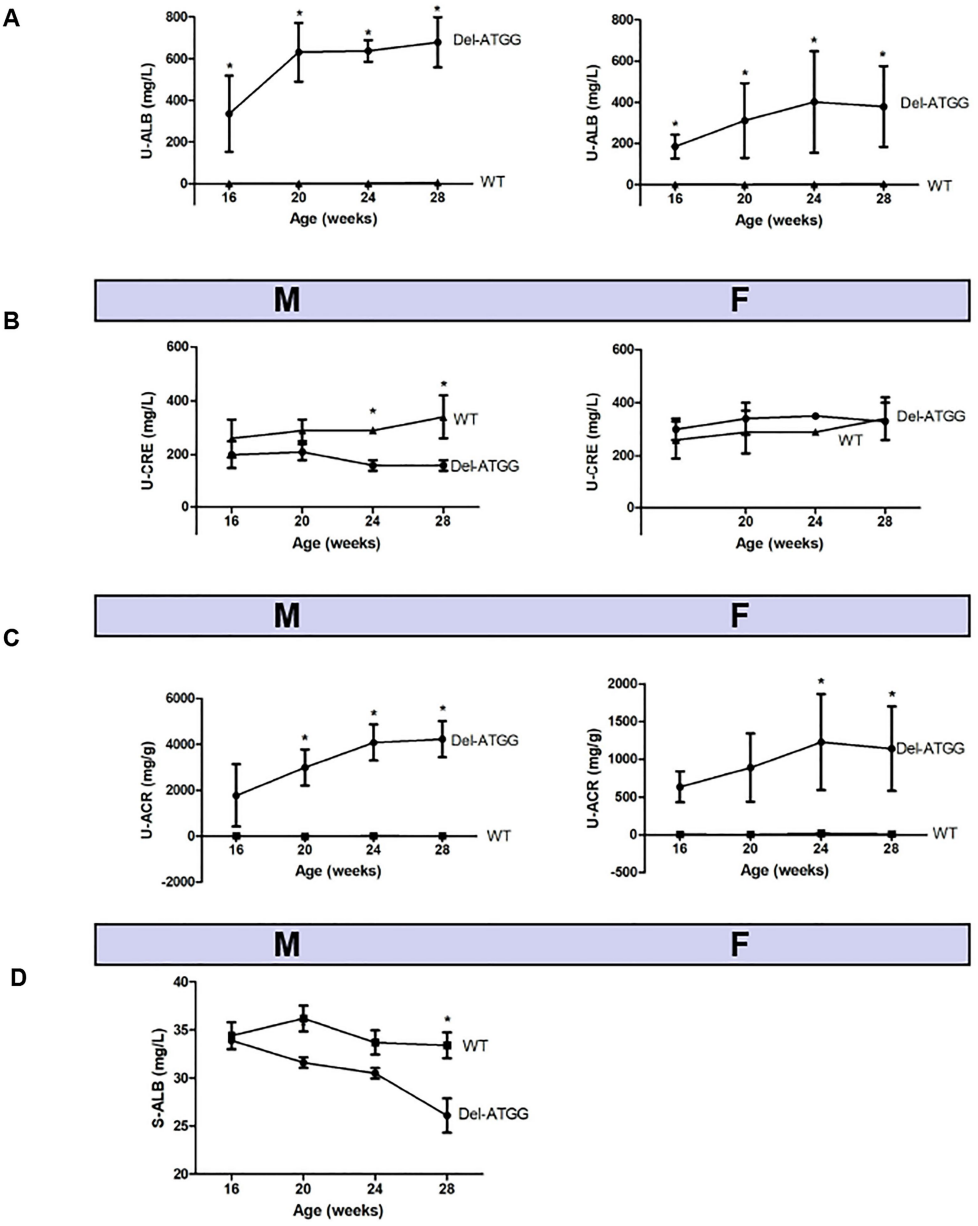
The biochemical parameters of Del-ATGG mice. **(A)** Albumin (ALB) alterations of 24-h urine. **(B)** Creatinine (CRE) alterations of 24-h urine. **(C)** Albumin-to-creatinine ratio (ACR) alterations in 24-h urine. **(D)** ALB alterations of serum. M, hemizygous male mice; F, heterozygous female mice, WT, wild-type mice. Asterisk indicates *t* significant difference of *p* < 0.05 between Del-ATGG male and female mice versus wild-type. Samples for urine biochemical test include 7 Del-ATGG hemizygous male mice, 11 Del-ATGG heterozygous female mice, and 6 normal genotype control mice. For serum biochemical test, it was 2 Del-ATGG hemizygous male mice and 3 control male mice.

Compared with wild-type mice, hemizygous male mice and heterozygous female mice had developed functional abnormalities of kidneys at 16 weeks. The urine ALB levels of Del-ATGG mice were significantly higher than wild-type mice with aging (Figure 3A). Since the ALB values of hemizygous male mice were higher than heterozygous female mice, the proteinuria of affected male mice was more severe. In contrast, serum ALB of Del-ATGG mice were gradually downregulated (Figure 3D). However, the urine CRE showed no difference between Del-ATGG and wild-type mice (Figure 3B). Only slightly decreased values of urine CRE were observed in hemizygotes. In contrast, Del-ATGG mice showed significantly higher values of urine ACR than wild-type after 20 weeks of age (Figure 3C). From 20 to 24 weeks of age, the values of ACR in Del-ATGG mice increased most significantly. And this tendency gradually slowed down from 24 to 28 weeks.

### 3.3. Histopathological alterations in Del-ATGG mice

**Figure 4:**
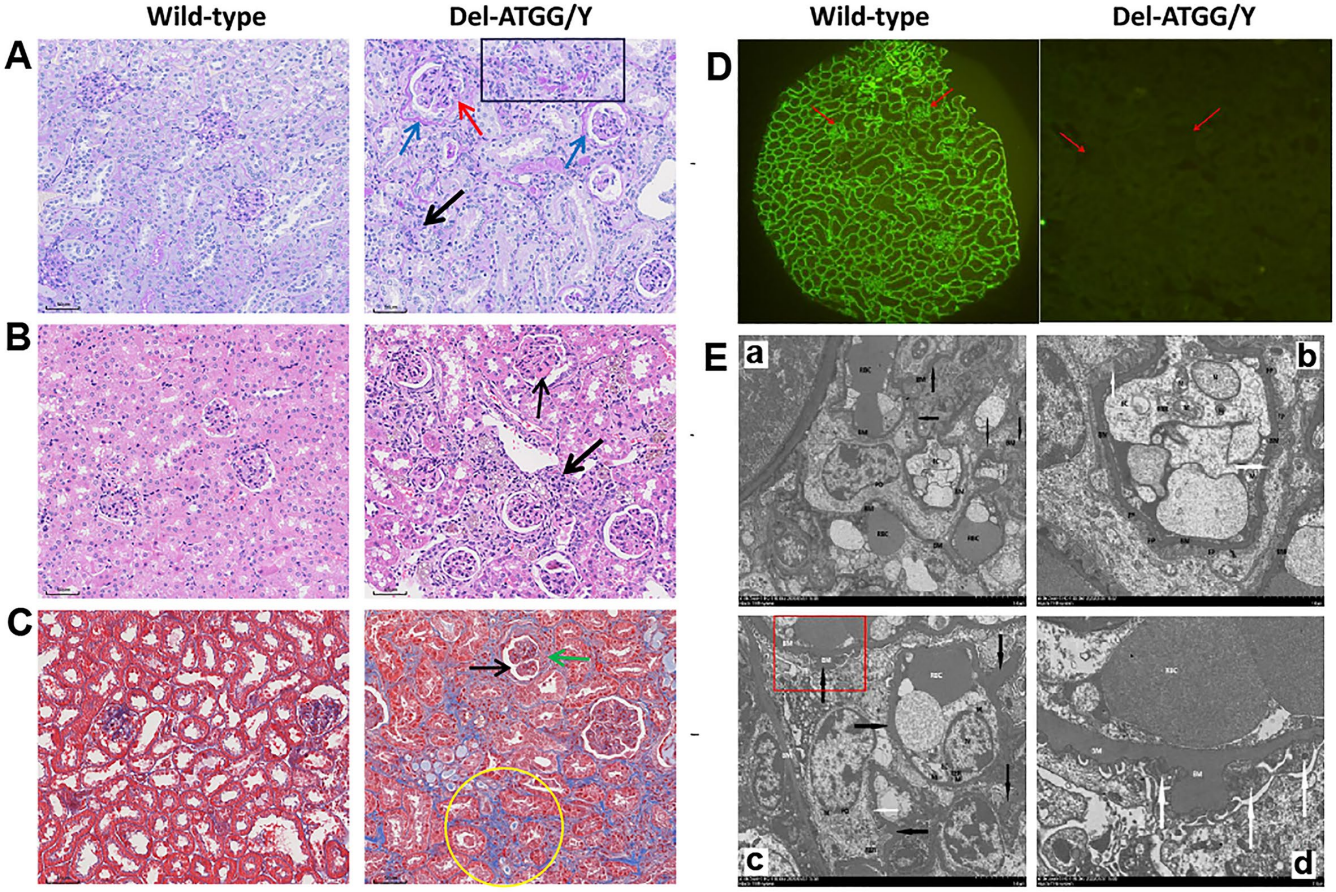
Histological alterations in kidney tissues of Del-ATGG mice. **(A–C)** The representative images of PAS, H&E, and Masson staining in kidney tissues from wild-type and Del-ATGG male mice at 30 weeks of age (magnification ×200). The black arrow indicated the widened mesangial area. The yellow arrow indicated the infiltration of mononuclear cells. The blue arrow indicated the increased and stratified parietal layer. The green arrow indicated the cellular crescentic body. The red arrow indicated the adhered loops. The black box indicated the atrophy and thickening renal tubule. The yellow circle indicated the focal fibrosis. **(D)** The immunofluorescence staining of type IV collagen α5 chain. The red arrow indicated the position of GBM. **(E)** The representative images of TEM in GBM from wild-type and Del-ATGG male mice at 30 weeks of age (Scale bar = 5 μm or 2 μm). The black arrows indicated basement membrane. The white arrows indicated the fusion or disappearance of foot processes. Figure D was the magnification of the structure in the red box of Figure C.

Compared with wild-type mice, the renal tissues of hemizygous male mice exhibited histopathological alterations. Until 30 weeks of age, the histological staining of hemizygous male mice showed some increased glomerulus volume, and glomerular atrophy and sclerosis. The mesangial area was mildly to moderately widened, some loops twisted and shrunken, and even individual loop adhered to the parietal layer. The thick of parietal layer was increased and stratified, and the cellular crescents were formed in some cysts. The tubular cells exhibited swelling, atrophy, and the lumen existed protein casts. Chronic interstitial lesions were also observed (Figures 4A–C). The expression of type IV collagen α5 chain was absent in glomerular capillary loop and Bowman’s capsule wall (Figure 4D).

Under the observation of the structure of GBM by TEM at 30 weeks mice, the GBM of wild-type male mice was clear, continuous, and uniform in thickness, with few areas occasionally thickened (Figure 4E). While, the GBM of Del-ATGG hemizygous male mice was irregular thinning, thickening, splitting with lamellation and local swelling. The foot process fused or disappeared in a large region, and the gap between foot processes was widened.

### 3.4. The type IV collagen alterations of Del-ATGG male mice

**Figure 5:**
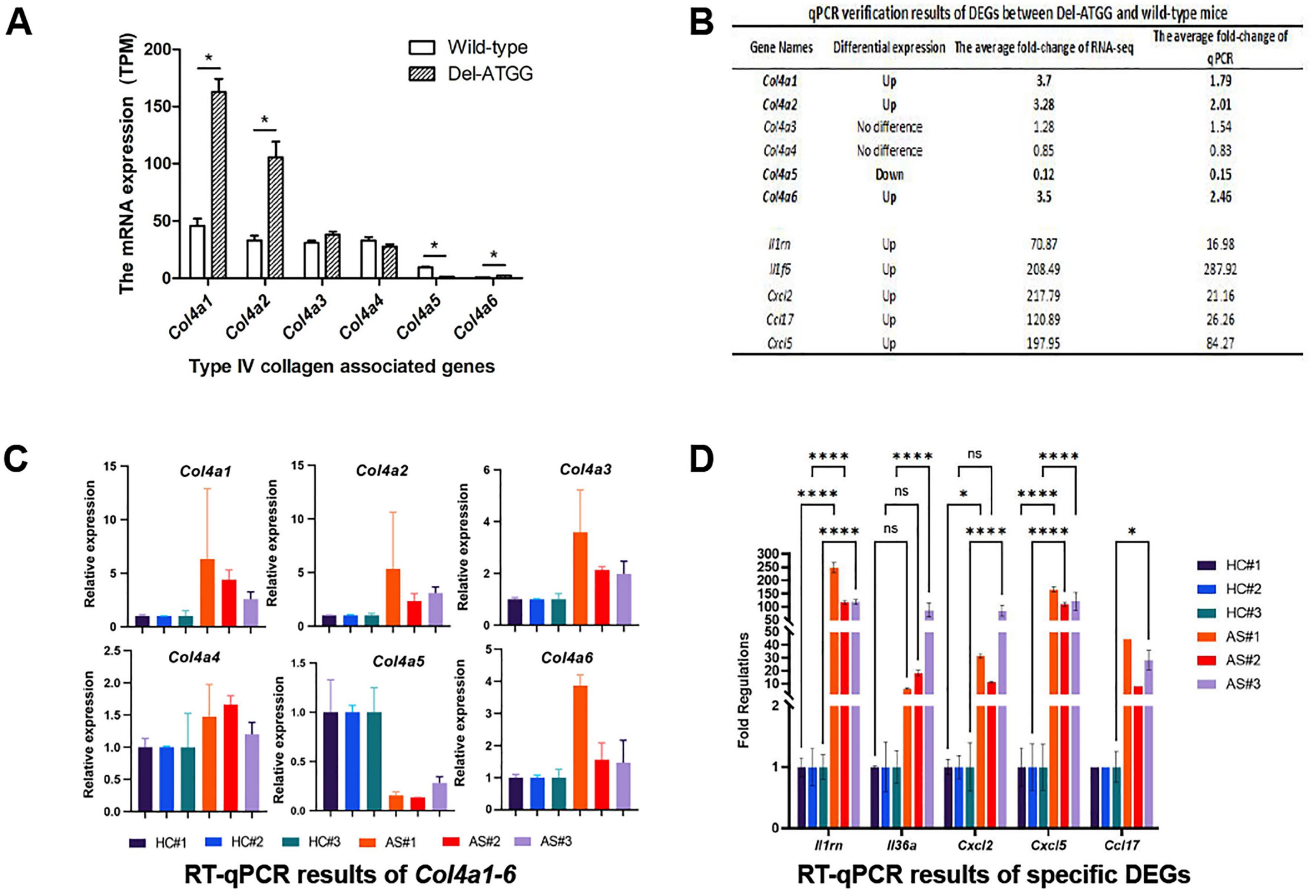
The expression of type IV collagen associated genes *Col4a1–6* and other DEGs in Del-ATGG mice. **(A,B)** The relative mRNA expression of *Col4a1–6* analyzed by RNA-seq. Each group contained 4 animals. **(B–D)** The qPCR verification results of DEGs between Del-ATGG and wild-type male mice, including *Col4a1–6*. Each group contained 3 animals.

RNA-seq results of renal tissues indicated that the expression of the *Col4a5* was significantly decreased in kidneys of Del-ATGG male mice, while the expressions of the *Col4a1* and *Col4a2* were compensatory increased (Figure 5A). The expressions of the *Col4a3* were increased and the *Col4a4* decreased slightly, but there was no statistically significant difference. Those alterations were verified by qPCR (Figure 5B). To verify the reliability of RNA-seq results, the *Col4a1–6* genes and a group of most significantly changed DEGs were selected for quantitative analyses. The results of qPCR were basically consistent with RNA-seq (Figures 5C,D).

The read counts of the *Col4a5* gene were between 0 and10 in Del-ATGG male mice and between 50 and 70 in wild-type mice (data not shown). There was no significant difference in the read counts of upstream and downstream region of mutation site, and a very low normalized expression of exon 17 was detected in Del-ATGG male mice.

### 3.5. The comprehensive transcriptomic alterations of Del-ATGG mice

**Figure 6:**
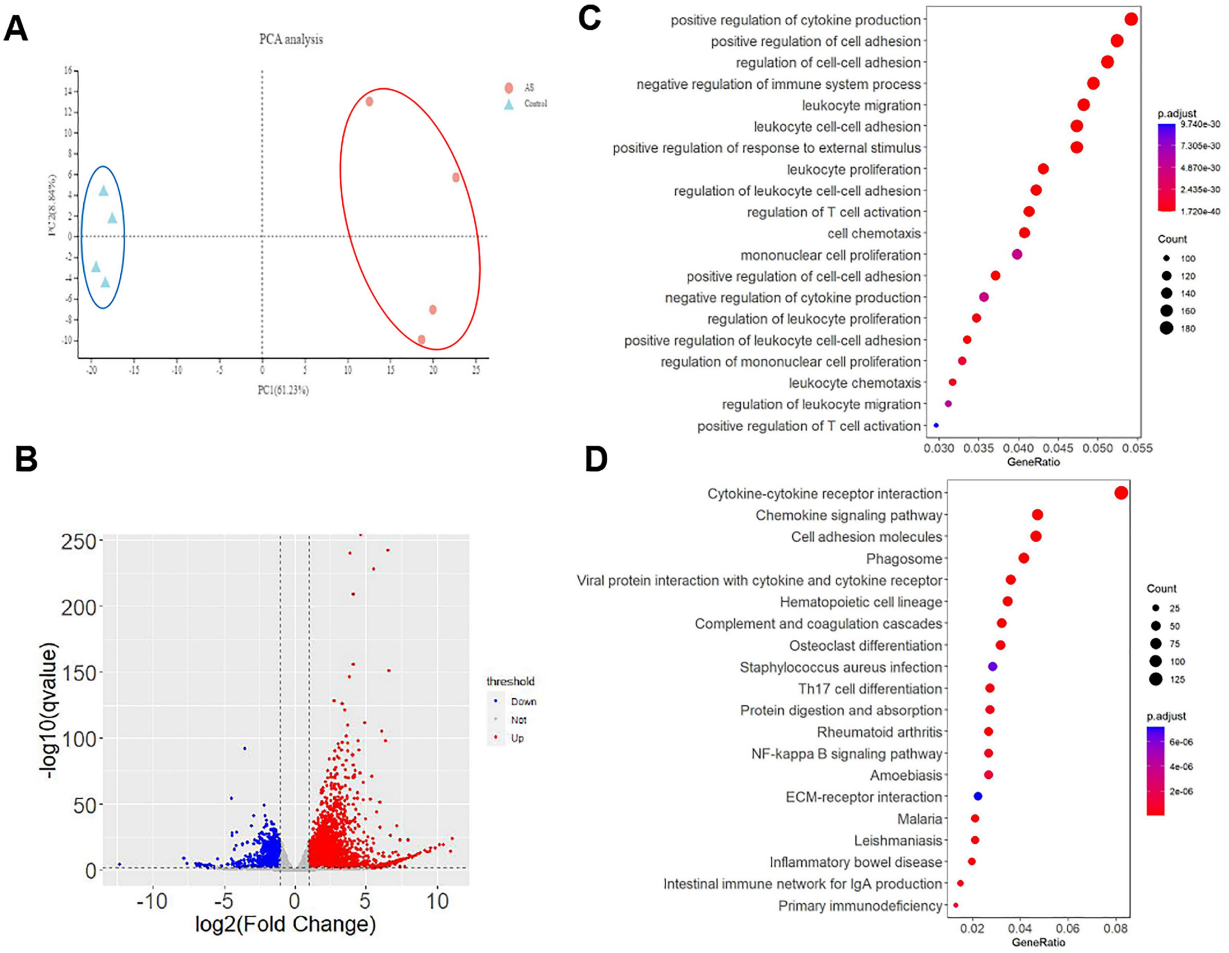
The transcriptomic alterations in Del-ATGG mice. **(A)** Principal component analysis (PCA) for all samples, the Del-ATGG male samples and control samples are clustered separately. **(B)** The volcano picture of DEGs between Del-ATGG and wild-type mice. The significantly differentiated genes in Del-ATGG were labeled. Red indicated significantly upregulated genes; blue indicated significantly downregulated genes. **(C,D)** GOBP and KEGG enrichment analysis result. The ordinate is the top 20 enriched pathways, the abscissa is the significant *p*-value of gene expression differences in the corresponding pathways. The size of the dots indicates the number of DEGs on this pathway.

The clean data of each sample were above 6.11 GB, the Q30 values of each sample were above 94.5%. The principal component analysis (PCA) clustered the four Del-ATGG male samples and the four control samples separately, showing good consistency within each group and significant difference between groups (Figure 6A).

In average, 24,003 genes were mapped in each sample, totally 4,188 DEGs (17.45%) were identified with *p*-value <0.05, including 2,826 upregulated and 1,198 downregulated genes (Figure 6B). The top 50 significant DEGs sorted by *p*-value are listed in Table 1, including 5 collagen genes (*Col3a1, Col4a5, Col15a1*, and *Col1a1*). All the DEGs are listed in Supplementary Table S2.

**Table 1:** Top 50 significant DEGs sorted by adjust *p*-value.

No.	Gene	Gene full name	Regulation	Log_2_ fold change	Adjust *p*-value
1	*C3*	Complement component 3	Up	25.42	3.42E-251
2	*Havcr1*	Hepatitis A virus cellular receptor 1	Up	95.62	3.47E-243
3	*Spp1*	Secreted phosphoprotein 1	Up	15.32	1.41E-240
4	*Serpina10*	Serine (or cysteine) peptidase inhibitor, clade A (alpha-1 antiproteinase, antitrypsin), member 10	Up	46.89	1.27E-228
5	*Vcam1*	Vascular cell adhesion molecule 1	Up	17.95	7.62E-210
6	*Cd44*	CD44 antigen	Up	17.91	2.57E-156
7	*Ltbp2*	Latent transforming growth factor beta binding protein 2	Up	98.04	1.54E-151
8	*Col3a1*	Collagen, type III, alpha 1	Up	14.58	3.31E-147
9	*Cdh11*	Cadherin 11	Up	6.90	8.70E-129
10	*Dpysl3*	Dihydropyrimidinase-like 3	Up	10.11	8.62E-127
11	*Mmp2*	Matrix metallopeptidase 2	Up	11.48	7.56E-122
12	*Sox9*	SRY (sex determining region Y)-box 9	Up	30.78	1.27E-112
13	*Ctss*	Cathepsin S	Up	13.16	2.41E-110
14	*Krt20*	Keratin 20	Up	68.86	7.41E-106
15	*Thbs2*	Thrombospondin 2	Up	12.24	7.04E-102
16	*Lyz2*	Lysozyme 2	Up	21.96	8.12E-99
17	*Timp1*	Tissue inhibitor of metalloproteinase 1	Up	85.77	2.52E-98
18	*D17H6S56E-5*	DNA segment, Chr 17, human D6S56E 5	Up	10.03	1.22E-97
19	*Lox*	Lysyl oxidase	Up	13.12	1.60E-96
20	*Slc7a2*	Solute carrier family 7 (cationic amino acid transporter, y+ system), member 2	Up	8.22	4.09E-96
21	*Mpeg1*	Macrophage expressed gene 1	Up	8.81	3.94E-93
22	*Ccl2*	Chemokine (C-C motif) ligand 2	Up	23.11	1.47E-91
23	*Smoc2*	SPARC related modular calcium binding 2	Up	9.93	3.02E-91
24	*Serpine2*	Serine (or cysteine) peptidase inhibitor, clade E, member 2	Up	7.10	1.00E-90
25	*Fgg*	Fibrinogen gamma chain	Up	13.47	1.22E-90
26	*Itgam*	Integrin alpha M	Up	16.77	5.95E-88
27	*Adcy7*	Adenylate cyclase 7	Up	8.00	7.11E-87
28	*Adgre1*	Adhesion G protein-coupled receptor E1	Up	8.60	2.34E-85
29	*Cp*	Ceruloplasmin	Up	6.84	4.42E-85
30	*Col15a1*	Collagen, type XV, alpha 1	Up	5.49	1.02E-84
31	*Col1a1*	Collagen, type I, alpha 1	Up	12.55	7.08E-84
32	*Aoc1*	Amine oxidase, copper-containing 1	Up	17.72	2.49E-80
33	*Aldh1a2*	Aldehyde dehydrogenase family 1, subfamily A2	Up	10.46	3.34E-80
34	*Fn1*	Fibronectin 1	Up	7.37	3.42E-78
35	*Mrc1*	Mannose receptor, C type 1	Up	11.87	1.02E-76
36	*Sulf1*	Sulfatase 1	Up	7.24	1.48E-76
37	*Mfap5*	Microfibrillar associated protein 5	Up	19.29	1.03E-75
38	*Sparc*	Secreted acidic cysteine rich glycoprotein	Up	4.93	1.35E-75
39	*Lif*	leukemia inhibitory factor	Up	24.45	6.92E-74
40	*Ccdc80*	Coiled-coil domain containing 80	Up	7.66	1.29E-73
41	*Mmp14*	Matrix metallopeptidase 14 (membrane-inserted)	Up	7.94	1.70E-73
42	*Abi3bp*	ABI family member 3 binding protein	Up	5.86	1.86E-73
43	*Dcdc2a*	Doublecortin domain containing 2a	Up	7.35	4.47E-73
44	*Siglec1*	Sialic acid binding Ig-like lectin 1, sialoadhesin	Up	18.65	1.05E-72
45	*Col1a2*	Collagen, type I, alpha 2	Up	7.03	6.68E-72
46	*Lgi2*	Leucine-rich repeat LGI family, member 2	Up	43.36	7.07E-72
47	*Serpine1*	Serine (or cysteine) peptidase inhibitor, clade E, member 1	Up	17.65	1.76E-71
48	*Dock10*	Dedicator of cytokinesis 10	Up	6.64	2.49E-71
49	*Runx1*	Runt related transcription factor 1	Up	12.08	2.69E-71
50	*Foxj1*	Forkhead box J1	Up	4.21	7.42E-71
1	*Col4a5*	Collagen, type IV, alpha 5	Down	−3.52	1.61E-92
2	Hba-a2	Hemoglobin alpha, adult chain 2	Down	−4.48	4.86E-55
3	Gnmt	Glycine N-methyltransferase	Down	−2.15	1.99E-49
4	Slc6a19	Solute carrier family 6 (neurotransmitter transporter), member 19	Down	−2.08	6.30E-42
5	Lrrc66	Leucine rich repeat containing 66	Down	−2.88	1.42E-41
6	Ass1	Argininosuccinate synthetase 1	Down	−1.98	2.14E-38
7	Sgk2	Serum/glucocorticoid regulated kinase 2	Down	−1.66	3.42E-37
8	Prodh	Proline dehydrogenase	Down	−1.60	2.12E-35
9	Gm5424	Argininosuccinate synthase pseudogene	Down	−1.99	6.01E-35
10	Isoc2a	Isochorismatase domain containing 2a	Down	−1.49	6.53E-35
11	Hpd	4-hydroxyphenylpyruvic acid dioxygenase	Down	−3.07	3.88E-34
12	Igfbp3	Insulin-like growth factor binding protein 3	Down	−1.98	7.59E-34
13	Degs2	Delta(4)-desaturase, sphingolipid 2	Down	−2.15	7.99E-33
14	Fxyd2	FXYD domain-containing ion transport regulator 2	Down	−1.45	8.07E-32
15	Klk1	Kallikrein 1	Down	−1.63	7.77E-31
16	Upb1	Ureidopropionase, beta	Down	−1.67	8.70E-31
17	Gabarapl1	Gamma-aminobutyric acid (GABA) A receptor-associated protein-like 1	Down	−1.36	9.58E-31
18	Mccc2	Methylcrotonoyl-Coenzyme A carboxylase 2 (beta)	Down	−1.40	2.10E-30
19	Sephs2	Selenophosphate synthetase 2	Down	−1.44	2.93E-30
20	Gstk1	Glutathione S-transferase kappa 1	Down	−1.36	5.05E-30
21	Cbs	Cystathionine beta-synthase	Down	−2.33	1.73E-29
22	Alas2	Aminolevulinic acid synthase 2, erythroid	Down	−4.07	2.77E-29
23	Hbb-bs	Hemoglobin, beta adult s chain	Down	−4.42	9.28E-29
24	Hmcn2	Hemicentin 2	Down	−2.33	2.97E-28
25	Insrr	Insulin receptor-related receptor	Down	−2.48	3.75E-28
26	Susd2	Sushi domain containing 2	Down	−1.96	4.43E-28
27	Slc22a18	Solute carrier family 22 (organic cation transporter), member 18	Down	−1.83	8.86E-28
28	Napsa	Napsin A aspartic peptidase	Down	−1.43	8.93E-28
29	Pink1	PTEN induced putative kinase 1	Down	−1.25	1.24E-27
30	Syn2	Synapsin II	Down	−2.17	1.53E-27
31	Pccb	Propionyl Coenzyme A carboxylase, beta polypeptide	Down	−1.18	1.53E-27
32	Selenbp1	Selenium binding protein 1	Down	−1.38	2.54E-27
33	Notum	Notum palmitoleoyl-protein carboxylesterase	Down	−1.31	2.57E-27
34	Hba-a1	Hemoglobin alpha, adult chain 1	Down	−4.42	1.32E-26
35	Fah	Fumarylacetoacetate hydrolase	Down	−1.46	2.76E-26
36	Fcgbp	Fc fragment of IgG binding protein	Down	−2.50	7.55E-26
37	Acy1	Aminoacylase 1	Down	−1.86	7.91E-26
38	Pxmp2	Peroxisomal membrane protein 2	Down	−1.37	8.22E-26
39	Trabd2b	TraB domain containing 2B	Down	−1.56	1.17E-25
40	Fmo1	Flavin containing monooxygenase 1	Down	−1.24	1.54E-25
41	Afmid	Arylformamidase	Down	−1.75	1.62E-25
42	Ahcy	S-adenosylhomocysteine hydrolase	Down	−1.43	2.10E-25
43	Ddt	D-dopachrome tautomerase	Down	−1.38	2.10E-25
44	Pcx	Pyruvate carboxylase	Down	−1.70	2.44E-25
45	Slc2a4	Solute carrier family 2 (facilitated glucose transporter), member 4	Down	−1.61	3.16E-25
46	Dpep1	Dipeptidase 1	Down	−1.43	4.04E-25
47	Nat8	N-acetyltransferase 8 (GCN5-related)	Down	−1.48	6.47E-25
48	Aamdc	Adipogenesis associated Mth938 domain containing	Down	−1.18	8.13E-25
49	Bckdha	Branched chain ketoacid dehydrogenase E1, alpha polypeptide	Down	−1.41	9.93E-25
50	Gstz1	Glutathione transferase zeta 1 (maleylacetoacetate isomerase)	Down	−1.14	1.11E-24

Gene Ontology (GO) analyses showed that totally 1,416, 79 and 140 statistically significant (*p*-value <0.05) GO-enriched terms were identified for biological processes, cellular components, and molecular function analyses, respectively. The top 20 significantly enriched pathways are shown in Supplementary Table S3. It was worth noting that cell adhesion, migration, proliferation, and related regulatory categories were prominently enriched in the biological process analyses (Figure 6C). Three cellular components directly related to the *Col4a5* gene, including collagen-containing extracellular matrix, collagen trimer, and basement membrane, were found in the top five significant categories in cellular component analyses (Supplementary Table S3). In the analyses of molecular functions, DEGs mainly involved in numerous biomolecular activity and binding.

As for Kyoto Encyclopedia of Genes and Genomes (KEGG) analyses, 81 significant enriched pathways were identified. In addition to enhanced degradation of ECM, quite a few pathways were related to amino acid metabolism, helper T-cell differentiation, and various receptor interactions. Besides, other enriched pathways included chemokine signaling pathway, NF-kappa B signaling pathway, JAK–STAT signaling pathway, TNF signaling pathway, PI3K-Akt signaling pathway, and Rap1 signaling pathway, indicating that the kidney of Del-ATGG mice in ESRD stage had significant pathological changes in immune responses, amino acid metabolism, cell survival, and cell adhesion. The top 20 significantly enriched pathways are shown in Supplementary Table S3 and Figure 6D.

## 4. Discussion

Alport syndrome (AS) is caused by mutations in *COL4A3*, *COL4A4* or *COL4A5* genes, mainly pathophysicalological alterations affect the kidney, but the pathophysiology of AS is still not fully understood. Several mouse models have been reported in the literature, which had play important role in exploring pathogenesis of AS (6). However, there were only two types of XLAS mouse models, carrying nonsense mutation G5X and R471X variant, respectively (7, 9), which to some extent limits research scope on pathological mechanism. The c.980_983delATGG of the *COL4A5* gene was a novel frameshift variant, reported in our previous study (14). The detailed clinical manifestations and renal and skin biopsy findings of the patient, presented in this article, were consistent with typical XLAS features. These evidences proved that Del-ATGG of the *COL4A5* gene is a pathogenic variant leading to XLAS. To understand the biochemical and pathogenic mechanisms of this type variant leading to the development of XLAS, a mouse model was constructed by CRISPR/Cas9 system. To the best of our knowledge, this is the first frameshift mutation XLAS model. Since the pathogenic mechanism of various variant types might be different, Del-ATGG mouse model provide a new tool and platform for the study of pathogenesis and therapeutic targets of XLAS.

Similar with the patient, Del-ATGG male mice showed hematuria, loss of expression of *Col4a5* in renal tissues, typical AS-like basement membrane alternation, and other histopathological findings of increased glomerular volume, mesangial hyperplasia, focal glomerular sclerosis, tubular atrophy, and interstitial fibrosis. These results proved that this *Col4a5* Del-ATGG XLAS mouse model could simulate the disease initiation and progression of human XLAS. In addition, glomerular crescents were noticed in Del-ATGG male mice, which was not observed in the patient, probably because the size of the patient’s renal tissue biopsy was limited. Crescents is an uncommon histological manifestation of AS, could be detected in 20% of AS patient, and occasionally found in other AS animal models, such as G471X mice model and autosomal dominant hereditary bull terrier AS model (16–18). But the formation mechanism of the crescents needs to be further studied.

According to the biochemical results, Del-ATGG male and female mice showed abnormal renal function and a rapid disease progression before 16 weeks of age, and progressed to extremely serious within 1 to 2 months. By 28 weeks, the hemizygous male mice progressed to ESRD, the longest survival time was 32 weeks of age. The onset time and survival time were consistent with the previous reported XLAS models of G5X and R471X (7).

Type IV collagen comprise six α chains (α1 to α6), which were encoded by *COL4A1-COL4A6* genes, forming three triple helix trimers, α1α1α2 (IV), α3α4α5 (IV), and α5α5α6 (IV). The normal mature GBM contains α3α4α5 (IV) and α1α1α2 (IV), while only α1α1α2 (IV) exists in GBM of XLAS male patient/mice, which was important trigger leading to renal disease in AS (19, 20). In this Del-ATGG male mice, the expression of *Col4a5* in mRNA level was reduced to less than 20% of wild-type male mice, while *Col4a1, Col4a2,* and *Col4a6* were significantly induced. This observation was consistent with that from an XLAS rat model with a nonsense variant, confirming again the theory that α3α4α5 (IV) trimer deficiency leads to the compensatory hyperplasia of α1α1α2 (IV), and also proving the success of XLAS model construction (8).

Nonsense or frameshift mutations containing premature stop codons (PTCs) may trigger NMD and lead to mRNA degradation, but some cases with those variants could escape NMD (21–23). According to the results of RNA-seq and qPCR, the mRNA expression of *Col4a5* of Del-ATGG mice was only 12–15% of the wild-type, no significant difference was noticed in the read counts of upstream and downstream region of Del-ATGG site, so we speculate that the low number of transcripts of the *Col4a5* gene was due to induced degradation of mRNA by NMD mechanism, while NMD escape did not occur. NMD is a nonspecific translation-dependent regulatory pathway in mammals, it had been suggested that the degradation of mRNA triggered by UPF1 was similar to a computing cloud providing a flexible infrastructure with rapid elasticity and dynamic access according to specific user needs (24). In this study, the expression levels of key NMD factors in Del-ATGG male mice, such as UPF1, SMG1, etc., were not found to be significantly different compared with the wild-type mice. Therefore, we speculated that the degradation of a single gene mRNA through NMD mechanism was not sufficient to cause significant changes in NMD-related molecules.

In this study, to comprehensively depict the pathological changes of Del-ATGG mice, 28-week-old Del-ATGG male mice were selected for DEGs analysis, which were close to the average death age and had entered the ESRD period of XLAS patients according to the results of urine biochemical examination and pathological detection. As far as we know, this is the first RNA-seq study of a XLAS mouse kidney tissue, with totally 4,188 DEGs between Del-ATGG and wild-type mice were identified, including 2,826 upregulated and 1,198 downregulated genes. Consistent with the previously two cases of transcriptomic research of XLAS canine model, this study also revealed the enriched pathways including bio-adhesion, T-cell activation, integrin-associated signaling pathways, inflammatory/immune responses, and matrix remodeling, but oxidative stress was not prominent as showed by previous researchers, which may due to different research methods and animal species (11, 12). AS had been noticed in the rapid group of XLAS Canine model, the “adhesion” in “biological process” terms was noteworthy, especially the “cell–cell adhesion,” revealing the functional changes such as cell proliferation, differentiation, migration, and apoptosis in the kidneys of XLAS mice, which suggesting that it is hopeful to identify important molecules or mechanisms involved in the occurrence and development of AS disease, if we focus on “adhesion” in the future research.

In conclusion, a Del-ATGG XLAS mouse model has been generated to present the biochemical, histological, and pathological alterations for evidence of pathogenicity of this frameshift variant. Further gene expression and transcriptome analyses confirmed a compensatory hyperplasia and revealed DEGs and enriched pathways likely related to the genetic modification on variable phenotypes of AS. This mouse model will facilitate further study of genetic and pathogenic mechanisms, clinical management, and therapeutic targets for AS.

## Data availability statement

The datasets presented in this study can be found in online repositories. The names of the repository/repositories and accession number(s) can be found in the article/Supplementary material.

## Ethics statement

The animal study was reviewed and approved by the ethics committee of Institutional Animal Care and Use Committee of Nanjing University School of Medicine.

## Author contributions

W-qW and J-xZ: performed the analysis, collected the clinical data of the patient, and draft manuscript preparation. Y-xC: study design and interpretation. M-cZ and C-hZ: Biochemical and interpretation of histopathological data. X-hC: revision of the manuscript. SD: preliminary RNA-sequencing analysis. P-nL: critical revision of the manuscript. X-jL: study design, coordination and interpretation of data. All authors contributed to the article and approved the submitted version.

## Funding

This work was supported by grants from the Science and Technology Program of Jiangsu Province (BL2014072), Shenzhen Science and Technology Innovation Committee (KJYY20180703173402020), and the National Key Clinical Program of China (2014ZDZK003).

## Conflict of interest

The authors declare that the research was conducted in the absence of any commercial or financial relationships that could be construed as a potential conflict of interest.

## Publisher’s note

All claims expressed in this article are solely those of the authors and do not necessarily represent those of their affiliated organizations, or those of the publisher, the editors and the reviewers. Any product that may be evaluated in this article, or claim that may be made by its manufacturer, is not guaranteed or endorsed by the publisher.
